# Characterization of Phenolic Compounds, Vitamin E and Fatty Acids from Monovarietal Virgin Olive Oils of “*Picholine marocaine*” Cultivar

**DOI:** 10.3390/molecules25225428

**Published:** 2020-11-19

**Authors:** Aziz Bouymajane, Yassine Oulad El Majdoub, Francesco Cacciola, Marina Russo, Fabio Salafia, Alessandra Trozzi, Fouzia Rhazi Filali, Paola Dugo, Luigi Mondello

**Affiliations:** 1 Team of Microbiology and Health, Laboratory of Chemistry-Biology Applied to the Environment, Faculty of Sciences, Moulay Ismail University, Zitoune Meknes BP 11201, Morocco; azizbouymajane.01@gmail.com (A.B.); fouzia.filali@yahoo.fr (F.R.F.); 2 Department of Chemical, Biological, Pharmaceutical and Environmental Sciences, University of Messina, 98168 Messina, Italy; youladelmajdoub@unime.it (Y.O.E.M.); fsalafia@unime.it (F.S.); alessandra.trozzi@unime.it (A.T.); pdugo@unime.it (P.D.); lmondello@unime.it (L.M.); 3 Department of Biomedical, Dental, Morphological and Functional Imaging Sciences, University of Messina, 98125 Messina, Italy; 4 Chromaleont s.r.l., c/o Department of Chemical, Biological, Pharmaceutical and Environmental Sciences, University of Messina, 98168 Messina, Italy; 5 Department of Sciences and Technologies for Human and Environment, University Campus Bio-Medico of Rome, 00128 Rome, Italy; 6 BeSep s.r.l., c/o Department of Chemical, Biological, Pharmaceutical and Environmental Sciences, University of Messina, 98168 Messina, Italy

**Keywords:** virgin olive oils, *Picholine marocaine*, phenolic compounds, vitamin E, fatty acids, HPLC-PDA/ESI-MS, NP-HPLC/FLD, GC-FID/MS

## Abstract

Olive oil is an important product in the Mediterranean diet, due to its health benefits and sensorial characteristics. *Picholine marocaine* is the most cultivated variety in Morocco. The present research aims to evaluate the phenolic compounds, vitamin E and fatty acids of commercial *Picholine marocaine* virgin olive oils (VOOs) from five different North Moroccan provinces (Chefchaouen, Taounate, Errachidia, Beni Mellal and Taza), using HPLC-photodiode array (PDA)/electrospray ionization (ESI)-MS, normal phase (NP)-HPLC/ fluorescence detector (FLD) and GC-flame ionization detector (FID)/MS, respectively. The obtained results showed an average content of 130.0 mg kg^−1^ of secoiridoids (oleuropein aglycone, 10-hydroxy-oleuropein aglycone and ligstroside aglycone, oleocanthal and oleacein), 108.1 mg kg^−1^ of phenolic alcohols (tyrosol and hydroxytyrosol), 34.7 mg kg^−1^ of phenolic acids (caffeic acid, ferulic acid and elenolic acid), and 8.24 mg kg^−1^ of flavonoids (luteolin, luteolin glucoside, apigenin). With regard to vitamin E, α-tocopherol was the most abundant vitamin E (57.9 mg kg^−1^), followed by α-tocotrienol (2.5 mg kg^−1^), γ-tocopherol (4.5 mg kg^−1^) and β-tocopherol (1.9 mg kg^−1^), while δ-tocopherol was not detected. Moreover, 14 fatty acids were found and, among them, oleic acid (76.1%), linoleic acid (8.1%) palmitic acid (8.7%) and stearic acid (2.5%) were the major fatty acids detected. Finally, heat map and principal component analysis allowed us to classify the studied provinces in terms of VOO chemical composition: Chefchaouen (tyrosol and hydroxytyrosol), Taounate (oleuropein aglycone), Errachidia (ferulic acid, *w*-3 and *w*-6), Beni Mellal (oleocanthal) and Taza (luteolin and oleic acid).

## 1. Introduction

In Morocco, olive cultivation represents a crucial socio-economic pillar, contributing up to 5% of agricultural gross domestic product and 15% of Moroccan agricultural foodstuff export [1]. The olive tree represents 65% (1,045,186 ha) of the tree growing area, of which 37% (384,528 ha) is irrigated, whereas 63% (660,658 ha) non-irrigated. Overall, 65% of olive production is destined for pressing, 25% for canning and 10% for losses and domestic consumption. In 2017, Morocco exports around 76% (70,000 tons) of table olive, 14% (128.00 tons) of olive pomace and 10% (88.00 tons) of olive oil [2]. Moreover, Moroccan olive oil exports are mainly intended for Spain, Portugal, Holland, Italy, the USA and Asia [2]. However, there is lack of an adequate database reflecting the quality and compositional peculiarities of olive oils. This could have negative repercussions for the commercialization of Moroccan olive oils due to non-compliance with the requirements of the international olive oil council [2].

**Figure 1:**
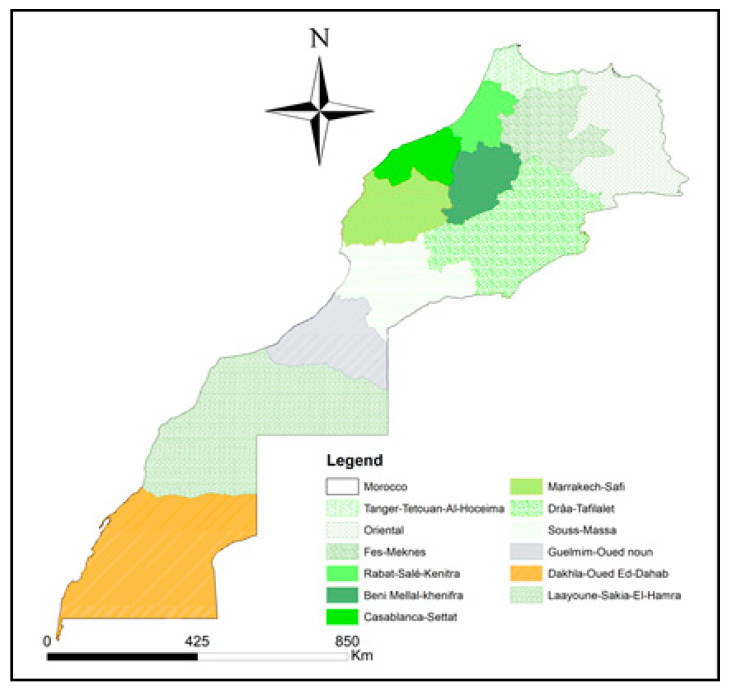
Political map of Morocco.

Olive oil extraction is carried out using three different extraction processes: (1) the traditional discontinuous press process; (2) the two-phase decanter process; (3) the three-phase decanter process. In the traditional discontinuous press process, the olives are picked, leaves are removed, olives are washed, crushed, kneaded with the warm water (38 °C) (paste) and pressed (olive pomace and olive mill wastewater) [3]. Then, the olive pomace is vertically centrifuged or decanted to obtain the oil. In the two-phase decanter process, the water naturally found in the olive is used to produce olive oil and olive mill waste (liquid and solid wastes) after centrifugation. In the three-phase decanter process, after beating, the water is added and the centrifugation is processed, producing olive oil, olive mill wastewater and olive solid mill wastes (olive cakes) [3,4]. Furthermore, in Morocco, the extraction of olive oil is done mainly using the two-phase decanter process. The main olive-growing regions are located in northern central Morocco: Fes-Meknes (346,000 ha), Marrakech-Safi (215,000 ha), Tanger-Tetouan Al-Hoceima (163,000 ha), Oriental (122,000 ha), Beni Mellal-Kenifra (80,000 ha), Rabat-Salé-Kenitra (66,135 ha), Souss-Massa (19,455 ha), Drâa-Tafilalet (16,000 ha), Casablanca-Settat (15,000 ha), and Guelmin-Oued noun (2000 ha) [2] (Figure 1). Moreover, within such regions, the provinces of Touanate, Taza, Chefchaouen, Beni Mellal and Errachidia have a stronger olive-growing vocation and occupy productive olive areas of 131.000 ha, 55.000 ha, 43.000, 14.000 ha and 3.000 ha, respectively [5].

The olive growing is dominated by *Picholine marocaine* cultivar (up to 96%), due to its high adaptability in bioclimatic stages (plains, mountainous, arid and Saharan area), organoleptic characteristics (medium green fruitiness, bitterness and balanced spiciness), the richness of the chemical and aromatic profiles and double purpose (production of olive oil and canned olive). Furthermore, the Green Moroccan Plan encourages the diversification of the varieties, including Dahbia, Haouzia and Menara varieties. Moreover, other Spanish and Italian varieties have been introduced (Picual, Frantoio, Manzanilla, Gordal, Arbequina, etc.).

Olive oil is a functional food containing a variety of components, which contribute to its overall sensory characteristics and health benefits. The nutritional value of olive oil can be attributed to its high added value bioactive substances, such as phenolic compounds, vitamin E, fatty acids, carotenoids and phytosterols [6].

Phenolic compounds are secondary metabolites that are widely distributed in plants, including flavonoids, isoflavonoids, phenolic acids, proanthocyanidins, tannins and lignans [7]. Phenolic compounds are contained in good amount in drupes, but during olive oil extraction over 90% of such molecules, due to their hydrophilic character, are distributed into pomace and mill wastewater [8,9,10]. The majority of these compounds are phenolic acids (e.g., hydroxytyrosol and tyrosol), secoiridoids (e.g., oleuropein) and lignans (e.g., pinoresinol) [11,12,13]. Phenolic compounds have been recognized due to claims about their health benefits, such as protection from low-density lipoproteins (LDL), maintenance of normal blood pressure and high-density lipoproteins (HDL) concentrations, anti-inflammatory properties, contribution to the upper respiratory tract health and maintenance of the normal function of the gastrointestinal tract [14]. Indeed, phenolic compounds contribute to the stability of olive oils against auto-oxidation and they are clearly involved in the organoleptic characteristics of olive oils such as bitterness, pungency and astringency [15,16,17,18]. Moreover, olive oil contains vitamin E, which is a lipophilic vitamin, including a mixture of four different forms of tocopherols (α, β, γ, δ) and four different forms of tocotrienols (α, β, γ, δ), with α-tocopherol being a dominant antioxidant constituent of vitamin E, playing a major role in scavenging reactive oxygen species (ROS) generated by the endogenous system, thus contributing the body’s defense system. α-tocopherol is involved in protection against the oxidation of polyunsaturated fatty acids (PUFAs) within membrane phospholipids (preservation of membrane integrity and stability, the stability of erythrocytes and conductibility of nerves, prevention of haemolytic anaemia and neurological symptoms; ataxia, peripheral neuropathy, myopathy and pigmented retinopathy) and plasma lipoproteins [19].

Olive oil is rich in triacylglycerol (99%), containing monounsaturated fatty acids (oleic acid, palmitoleic acid), saturated fatty acid (palmitic acid) and polyunsaturated fatty acids (linoleic acid). It contains smaller amounts (1%) of free fatty acids, monoglycerides, diglycerides, phosphatides, waxes, and sterol esters [20]. The chemical composition of olive oil depends on a set of factors such as the olive cultivar (characteristics), agronomics (irrigation and fertilization), cultivation practices (harvesting and maturity), technological specifications (storage and extraction system), and geographical features (altitude, latitude, edaphological characteristics) [21,22,23,24,25].

The objective of this study was to elucidate the relationship between the chemical composition of virgin olive oils (VOOs) of *Picholine marocaine* and the studied provinces, namely Chefchaouen, Taounate, Errachidia, Beni Mellal and Taza. For this purpose, phenolic compounds, vitamin E and fatty acids were analyzed using liquid chromatography coupled with a photodiode array and electrospray ionization mass spectrometry (HPLC-PDA/ESI-MS), a normal phase-high performance liquid chromatography/fluorescence detector (NP-HPLC/FLD) and gas chromatography coupled with both mass spectrometry and a flame ionization detector (GC-FID/MS), respectively.

## 2. Results and Discussion

In this work, phenolic compounds, vitamin E and fatty acids in the VOOs of *Picholine marocaine*, obtained from samples belonging to five different provinces of Morocco (Taounate, Errachidia, Chefchaouen, Beni Mellal and Taza), were identified and quantified as indicated in Table 1 and Table 2.

Being commercial samples, the acidity of the investigated VOOs was evaluated according to [26]. All of them can be classified as virgin with an acidity range from 1.1 to 2.0%.

All analyzed samples revealed α-tocopherol as the most abundant vitamin E (ranging from 38.38 mg kg^−1^ to 100.36 mg kg^−1^), whereas δ-tocopherol was below the limit of quantification (LOQ) in all samples. The Taounate sample of the Fes-Meknes region presented the richest value of vitamin E (107.63 mg kg^−1^); on the other hand, the poorest one was found for the Taza province, belonging to the same region (46.07 mg kg^−1^). The highest content of α-tocotrienol was detected in Beni Mellal (7.60 mg kg^−1^); on the other hand, for Taounate and Errachidia, the α-tocotrienol content was below the LOQ. The qualitative and quantitative profiles of vitamin E in Errachidia and Chefchaouen are very similar to one another. Such a finding is consistent with a previously published paper on Moroccan, Tunisian and Spanish olive oils, where α-tocopherol and γ-tocopherol were reported at a concentrations of 30 mg kg^−1^ and 5 mg kg^−1^, respectively, whereas γ-tocopherol was not detected [30]; also, the attained values for α-tocopherol in the VOO samples fall within the average content recommended by the United States Department of Agriculture (USDA) [31].

In our study, different classes of phenolic compounds were characterized in VOO, including phenolic alcohols (tyrosol and hydroxytyrosol), phenolic acids (caffeic acid, ferulic acid and elenolic acid), secoiridoids (oleuropein aglycone, 10-hydroxy-oleuropein aglycone and ligstroside aglycone, oleocanthal and oleacein) and flavonoids (luteolin, luteolin glucoside, apigenin), all of which were positively identified. As can be seen in Table 1, the highest phenolic content was found in VOO from Chefchaouen (723.47 mg kg^−1^), whereas the lowest was recorded for the Errachidia (17.99 mg kg^−1^). The ranges for all regions were as follows: phenolic alcohols (<LOQ-340.6 mg kg^−1^), phenolic acids (13.06–89.55 mg kg^−1^), secoiridoids (3.86–314.04 mg kg^−1^) and flavonoids (1.07–17.96 mg kg^−1^). In terms of secoiridoids, the highest amount was found in VOOs from Taounate (314.04 mg kg^−1^) and Chefchaouen (285.39 mg kg^−1^). Furthermore, the highest phenolic acid concentration in VOOs from Chefchaouen was found at 89.55 mg kg^−1^, whereas the highest flavonoid concentration was found in VOO from Beni Mellal (17.96 mg kg^−1^) followed by Taza (11.69 mg kg^−1^). Samples belonging to Fes-Meknes regions, despite the fact that they come from the same area, showed a very different content of bioactive molecules, e.g., the content in vitamin E samples from Taounate were more than double that in the Taza sample, whereas the phenolic content was seven times higher. This could be due to bottling or storage conditions that could have affected the VOO acidity and consequently the bioactive molecule content. The quantitative variation in phenolic compounds observed may be due to the environmental conditions (temperature, pH, moisture, soil, microorganisms etc.), harvesting method, fruit ripeness, extraction process or storage, as previously reported [32,33,34]. The findings of this study are in agreement with previous ones with the highest secoiridoid concentration and the lowest flavonoid and phenolic alcohol concentrations (except Taounate province) [35]. The values attained for tyrosol in VOOs coming from Chefchaouen (300.57 mg kg^−1^) and Beni Mellal (186.17 mg kg^−1^) are much higher with respect to the mean value for commercial extra virgin olive oils (EVOOs) belonging to the Italian and European markets (56 mg kg^−1^) [36] This can be justified by the hydrolysis of oleuropein and oleacine [13]. These values are not in agreement with a previous work by Bajoub et al., who reported that a mean concentration of tyrosol in VOOs of *Picholine marocaine* from Chefchaouen, Taounate and Taza provinces ranged from 4.43 mg kg^−1^ to 7.46 mg kg^−1^ [35]. Similar considerations can be made for hydroxytyrosol content since Bajoub et al. reported a concentration range from 5.10 to 5.86 mg kg^−1^, differing from the values found in this work (<LOQ-40.03 mg kg^−1^). With regards to secoiridoids, oleuropein aglycone (1.94–231.34 mg kg^−1^), ligstroside aglycone (0.72–57.27 mg kg^−1^) and 10-hydroxy-oleuropein (0.26–65.73 mg kg^−1^) were found in the highest amount, whereas the lowest amount was attained for oleacein (0.20–33.27 mg kg^−1^) and oleocanthal (<LOQ-0.76 mg kg^−1^). According to Bajoub et al., the concentration value of secoiridoids in VOOs of *Picholine marocaine* from Meknes province ranged from 504.12 to 1106.96 mg kg^−1^ during the crop season of 2011–2013 [37]. Furthermore, oleuropein aglycone was found at the highest concentration in VOO of *Picholine marocaine* from Chefchaouen and Taounate and the lowest concentration in VOO from Errachidia. Ligstroside aglycone was found at the lowest concentration in VOO from Errachidia, whereas the highest concentration was found in VOO from Taounate [35]. Among the phenolic acids, elenolic acid was the most abundant one with a total concentration ranging from to 8.7 to 85.6 mg kg^−1^: the highest concentration was found in VOO from Chefchaouen province (85.61 mg kg^−1^), followed by Taounate province (31.28 mg kg^−1^) and Beni Mellal province (19.65 mg kg^−1^). Another study performed on VOO of *Picholine marocaine* showed that the total concentration of phenolic acids ranged from 0.13 to 0.22 mg kg^−1^ according the crop season (2011/2012) [38]. Bajoub et al. reported the highest concentration of elenolic acid in VOO from Taounate whereas the lowest concentrations were found in Chefchaouen and Taza [35]. Concerning flavonoids, the concentration of luteolin and apigenin was lower than the LOQ to a maximum of 7.80 mg kg^−1^ and lower than the LOQ to a maximum of 6.09 mg kg^−1^, respectively. These data are in agreement with recent studies performed on VOO of *Picholine marocaine*, reporting average concentrations of luteolin and apigenin of 3.20 mg kg^−1^ and 7.5 mg kg^−1^, respectively, from Errachidia province and Meknes region [30,38].

As far as fatty acid methyl esters are concerned, 14 fatty acid methyl ester extractions (FAMEs) were characterized in VOO using GC-FID/MS, as can be seen in Table 2. All the VOOs presented a similar qualitative profile, while some differences were found in the percentage composition of the identified FAMEs. Among them, oleic acid was the most abundant one, ranging from 69.79% to 79.39%, followed by palmitic acid (7.76–9.99%), and linoleic acid (6.25–12.67%). For oleic acid, Taza province recorded the highest value (79.39%), followed by Beni Mellal (78.15%), Chefchaouen (76.94%), Taounate (75.89%) and Errachidia (69.79%). In a previously published study on VOOs of *Picholine marocaine* from two provinces of Morocco (Errachidia and Marrakech), the lowest percentage of oleic acid and the highest of palmitic acid and linoleic acid were reported [39]. In the present study, the VOO coming from Errachidia province showed a content of oleic and linoleic acids that was slightly different compared to the other analyzed VOOs. Linoleic acid presented a concentration of 12.67%, which is roughly double in comparison with the other VOOs; on the other hand, oleic acid was 10% less in comparison with the VOO with the highest content, *viz.* Taza. Such variations across regions could be largely due to crop season (climate), maturity stage and geographic province, e.g., the desertic area where the olive trees grow [40].

**Figure 2:**
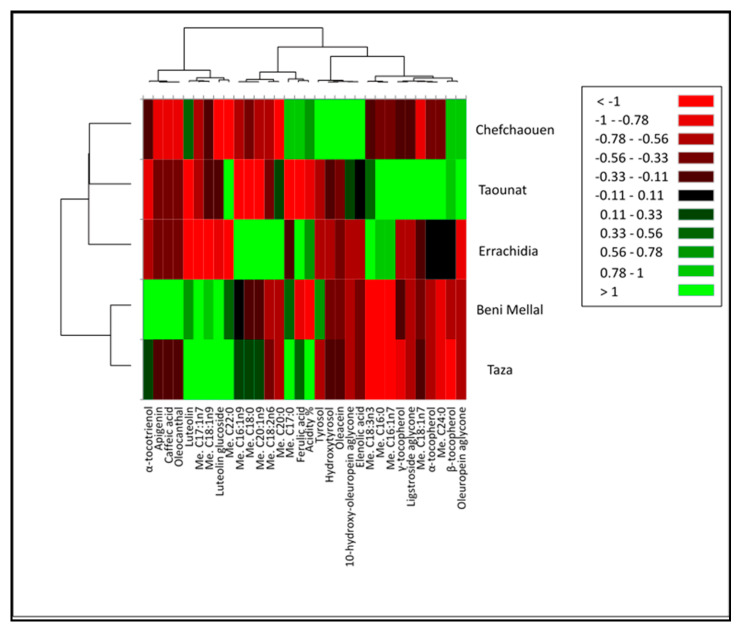
Heat map showing the distribution and concentration of phenolic compounds, fatty acids and vitamin E in VOO of *Picholine marocaine* from five Moroccan provinces. Green boxes mean a concentration higher than the mean value among the studied samples. A red box means lower concentrations.

In order to provide a more intuitive VOO chemical composition among the five different provinces studied, a heat map was drawn up (Figure 2). The considerations previously reported are clearly highlighted. Tyrosol, hydroxytyrosol, oleacein, elenolic acid and 10-hydroxytyrosol oleuropein aglycone classify the sample belonging to Chefchaouen; α- and β-tocopherols, fatty acids, e.g., palimitic (C16:0), cis-vaccenic acid (C18:1n7), behenic (C22:0) and lignoceric (C24:0) characterize the Taounate province. Errachidia can be classified with fatty acids, e.g., hypogeic (C16:1n9), stearic (C18:0), eicosenoic (C20:1n9), linoleic (C18:2n6), arachidic (C20:0) and α-linoleic (C18:3n3) and ferulic acid. The bioactive molecules that distinguish Beni Mellal province include apigenin, caffeic acid, oleocanthal, luteolin glucoside and α-tocotrienol. Finally, Taza province can be classified by considering luteolin and luteolin glucoside, oleic (C18:1n9) and margaric acids (C17:0).

**Figure 3:**
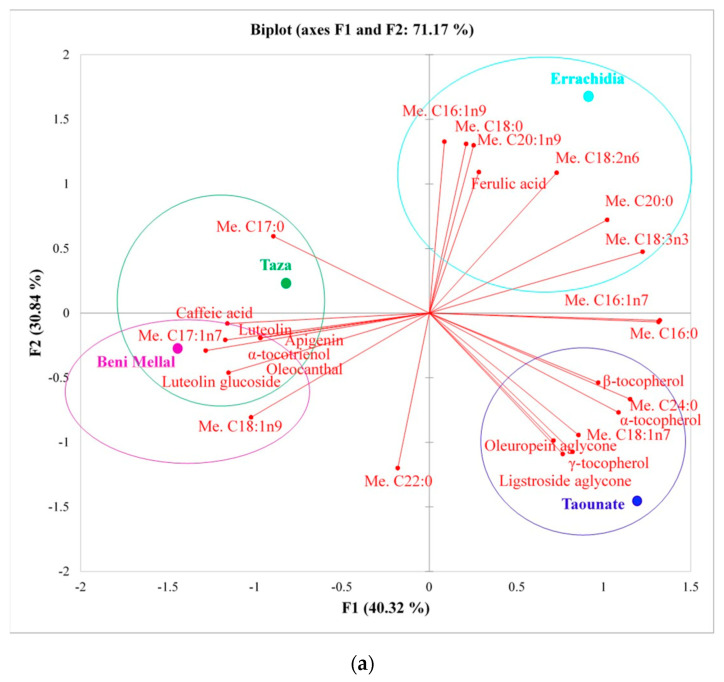

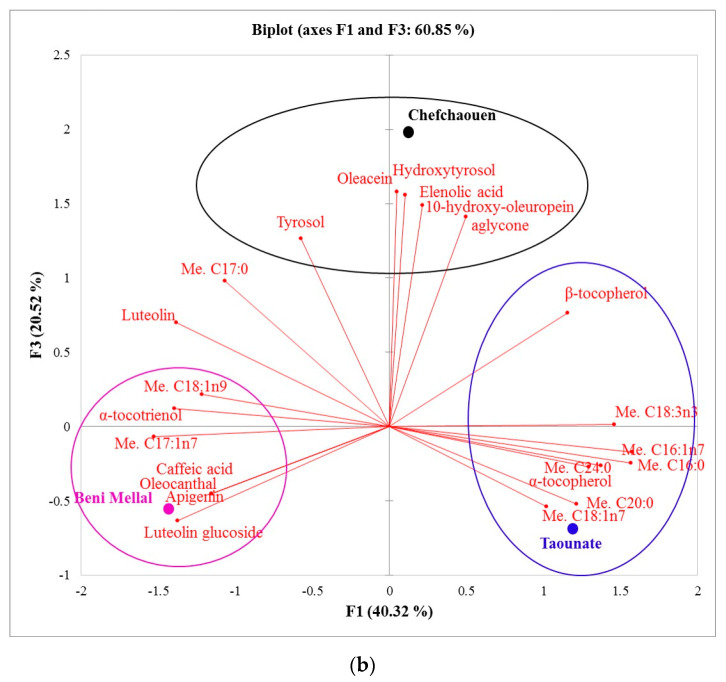
Principal component analysis (PCA) between phenolic compounds, vitamin E and fatty acids in VOO of *Picholine marocaine* and five provinces studied. (**a**) The first and second discriminant function; (**b**) the first and third discriminant function.

The use of such a heat map is undoubtedly a classification method that can be considered robust, selective and specific. With the employment of such a promising statistical method, it is possible to build a model for sample classification. Following an approach employed in a recent study and applied to eight cultivars [40], a principal component analysis (PCA) was applied to phenolic compounds, vitamin E and fatty acids in all VOO samples studied in order to identify the relationships between the explanatory variables. The first two principal components (F1 and F2) explained 71.2% of the variability, whereas F1 and F3 showed a value of 60.8%. The results achieved by PCA are similar to the ones attained by the heat map. As can be seen from Figure 3a, a positive correlation can be appreciated for Taza and Beni Mellal; on the other hand, in both Figure 3a,b, a negative correlation can be observed for Beni Mellal and Taounate. Moreover, for Errachidia–Beni Mellal, Chefchaouen–Tauonate, Chefchaouen–Beni Mellal, a negative correlation is highlighted by Figure 3a,b. Moreover, Figure 3a shows that Errachidia province has no correlation with Taounate. On the basis of the PCA results, each region presents a characteristic chemical pattern of bioactive molecules showing only one positive correlation between Taza province with Beni Mellal region. It is worth noting that within the same region, *viz.* Fes-Meknes, samples belonging to Taza and Taounate provinces show a negative correlation in the PCA, as also reported in Table 1.

## 3. Materials and Methods

### 3.1. Chemicals and Reagents

Commercial phenolic standards such as gallic acid (purity ≥97.5%), caffeic acid (≥98%), ethyl gallate (≥96%), luteolin (≥97%), oleuropein (≥98%), vanillic acid (≥95%), apigenin (≥99%), hydroxytyrosol (≥90%) and tyrosol (≥95%) were purchased from Merck Life Science (Merck KGaA, Darmstadt, Germany). Acetonitrile, water, *n*-hexane, methanol and formic acid were all HPLC grade and were purchased from Merck Life Science (Merck KGaA, Darmstadt, Germany). Diethyl ether, sodium hydroxide, sodium methanolate methanol, boron trifluoride, n-heptane and phenolphthalein were obtained from (Merck KGaA, Darmstadt, Germany). Tocopherols (α, β, γ, δ) and tocotrienol (α, β, γ, δ) standards were obtained from Extrasynthase (GenayCedex, France).

### 3.2. Sample Collection

VOO samples of the *Picholine marocaine* cultivar were purchased in a local market and belong to four different regions of Northern Morocco: Fes-Meknes (Taounate and Taza), Drâa-Tafilalet (Errachidia), Tanger-Tetouan-Al Hoceima (Chefchaouen) and Beni Mellal-Khenifra (Beni Mellal) [41]. According to the label reported, olives were harvested between October and December 2018.

### 3.3. Determination of Acidity

Five grams of VOO was dissolved in a mixture of diethyl ether and ethanol solvents (50:50, *v*/*v*). Then, 50 mL of titrated sodium hydroxide 0.1 M was used, with the addition of phenolphthalein as an indicator [26]. Acidity is expressed as follows:

Acidity (%) = 100 (M × V × M’/m)

where: M: molarity of sodium hydroxide solution (0.1 M); V: volume of titrated sodium hydroxide solution (mL), m: the weight of olive oil (g); M’: the molar weight of oleic acid (282 g/mol).

### 3.4. Phenolic Compounds Extraction

The extraction of phenolic compounds from VOO was determined following a protocol described previously [36,41,42]. Briefly, 1 g of VOO was dissolved in 1 mL of *n*-hexane, and then hexane phase was discarded. Then, 1 mL of a mixture of methanol:water (60:40, *v*/*v*) was added, vortexed, sonicated with ultrasound bath (rt, 37; Hz, 60 W) and centrifuged for 5 min at 3000 relative centrifugal force. The procedure was repeated three times. The extract was then dissolved in 500 µL of a mixture of methanol:water (60:40, *v*/*v*) and filtered through a 0.45 µm pore size membrane filter. Before injection to HPLC-PDA-ESI-MS, 20 µL (1000 ppm) of ethyl gallate, as an internal standard, was added to each extract.

### 3.5. HPLC-PDA-ESI-MS Analysis of Phenolic Compounds

HPLC analyses were performed using a Shimadzu instrument (Kyoto, Japan), composed of binary solvent pumps (LC-20AD), an SPD-M20A photodiode array and an LCMS-2020 mass spectrometry (MS) detector. The latter was equipped with an electrospray ionization (ESI) source operating in negative ionization (NI) mode. Data acquisition was conducted using LabSolution Ver. 5.91 software (Shimadzu, Kyoto, Japan).

The separation of phenolic compounds was carried out on an Ascentis Express C18 (150 × 4.6 mm, 2.7 µm) analytical column (Merck Life Science, Merck KGaA, Darmstadt, Germany). Mobile phases were as follows: A (H_2_O/0.1% HCOOH) and B (acetonitrile/0.1% HCOOH); the flow rate was 1 mL/min. The elution gradient was: 0.1 min 10% B, 4 min 35% B, 12 min 47% B, 12.5 min% B, min 60% B,16 min 75% B, 21 min 100% B and the injection volume was 10 µL. ESI-MS was performed with the following optimized parameters: capillary temperature 400 °C, capillary voltage 3500 V, nebulizer N_2_ pressure 45 psi, drying N_2_ flow rate 12 L/min, mass scan range (*m*/*z* 100–1000); the volume injection was 200 µL.

The chromatographic method was validated by determining the linearity, repeatability and recovery of the extraction at two fortification levels; limits of detection (LODs) and limits of quantification (LOQs) were estimated on the basis of 3:1 and 10:1 (signal-to-noise ratio). The calibration curves of the gallic acid, hydroxytyrosol, tyrosol, caffeic acid, oleuropein, luteolin and apigenin were constructed by injecting 5 μL of standard solution five times at four different concentrations (1 mg L^−1^, 25 mg L^−1^, 50 mg L^−1^ and 100 mg L^−1^) into the HPLC configuration. Derivates and isomers of oleuropein, elenolic acid and oleacein were quantified with the calibration curve obtained, using oleuropein as a standard. Verbascoside isomers and acetoxypinoresinol were quantified with the calibration curve of hydroxytyrosol. Tyrosol glucoside and ligstroside aglycone were quantified with the calibration curve of tyrosol.

### 3.6. NP-HPLC/FLD Analysis of Tocopherols and Tocotrienols

HPLC analyses were carried out using a Shimadzu Nexera-X2 system (Shimadzu, Kyoto, Japan) consisting of an online degasser (DGU-20ASR), an autosampler (SIL-30 AC), two dual-plunger parallel-flow pumps (LC-30AD), a column oven (CTO-20AC), and a fluorescence detector (RF-20AXS). Tocopherols (α, β, γ, δ) and tocotrienols (α, β, γ, δ) were separated on an Ascentis Si column (250 × 4.6 mm I.D. with 5 µm particle sizes, Merck Life Science, Merck KGaA, Darmstadt, Germany). VOOs were diluted in *n*-hexane (1:15 or 1:50) prior to being injected (5 µL) into the normal phase high-performance liquid chromatography (NP-HPLC) system coupled with fluorimetric detection (at 290 nm for the excitation wavelength and 330 nm for the emission wavelength). A mobile phase *n*-hexane-isopropanol was chosen and run in isocratic mode (99:1, *v*:*v*), while the flow rate was 1.7 mL/min. Data acquisition was conducted using LCMSsolution Ver. 5.85 software (Shimadzu, Kyoto, Japan).

### 3.7. Fatty Acid Methyl Esters Extraction (FAMEs)

Ten mg of olive oil was added to 500 µL of sodium methanolate methanol (0.5%, *w*/*v*). Then, the solution was mixed for 2 min at 2000 rpm and heated for 15 min at 95 °C. Subsequently, 50 µL of boron trifluoride diluted in methanol (14% *w*/*v*) was added to the reaction mixture and the solution was mixed for 2 min at 2000 rpm and heated for 15 min at 95 °C. After cooling, 350 µL of n-heptane and 300 µL of a saturated NaCl solution were added to the solution, and processed in a vortex at 2000 rpm for 2 min. Finally, the n-heptane FAME layer was taken and injected into the gas chromatographic systems.

### 3.8. GC–MS/FID Analysis of FAMEs

GC–MS analyses were carried out on a GCMS-QP2010 (Shimadzu, Duisburg, Germany) equipped with a split/splitless injector and AOC-20i autosampler. The chromatographic separation was performed on an SLBII60i capillary column (30 m × 0.25 mm id, 0.20 µm film thickness) (Merck Life Science, Merck KGaA, Darmstadt, Germany). The column oven temperature program ranged from 50 to 280 °C at a rate of 3 °C/min. The injection temperature was set at 280 °C/min, with an injection volume of 0.2 µL, using a split ratio of 1:50. Then, helium was used as a carrier gas with a linear velocity of 30 °C/cm and an inlet pressure of 26.6 kPa. For mass spectra analysis, a mass range of 40–550 *m*/*z*, electron ionization of 70 eV, an ion source temperature of 250 °C, an interface temperature of 200 °C and a detector voltage of 0.98 kV were used. FAMEs were identified by linear retention indices (LRIs) calculation, using C4–C24 standard solution. MS similarity spectra (over 90%) and LRI compared to the database values of lipids (LIPIDS Mass Spectral Library, Shimadzu, Kyoto, Japan) were used for peak assignments.

GC-FID analyses were carried out using a GC-2010 (Shimadzu, Duisburg, Germany) instrument equipped with a split/splitless injector, AOC-20i autosampler and FID detector. The column and temperature program was the same as that described for the GC–MS analysis. The FID parameters were as follows: FID temperature at 280 °C, hydrogen flow at 40 mL/min, make-up flow (nitrogen) at 30mL/min and air flow at 400 mL/min. All experiments were performed in triplicate.

### 3.9. Statistical Analysis

The results were expressed as mean values ± standard deviation (SD). All data were subjected to principal component analysis (PCA) and were included in a heat map. Olive oil origins (provinces) were considered as the variables in these plots and the different biological compounds (phenolic compounds, vitamin E, and fatty acids), except for the acidity, as treatments. PCA was applied to examine the relationship between the studied provinces and different biological compounds.

## 4. Conclusions

In this study, phenolic compounds, vitamin E and fatty acids of *Picholine marocaine* VOO from different provinces of Morocco were characterized by HPLC-PDA/ESI-MS, NP-HPLC/FLD and GC–MS/FID, respectively. *Picholine marocaine* VOOs of the studied provinces showed a variable amount of phenolic compounds such as tyrosol, hydroxytyrosol and oleuropein aglycone, as well as vitamin E. According to the International Regulations in force, the phenol content of three of them, namely Taounate, Chefchaouen and Beni Mellal, reached the established limits [27]; only one, *viz.* Taounate, complied with Regulation 432/2012 of the European Union in terms of vitamin E [28], whereas all of them satisfied the limits contained in COI 2001 for fatty acid content [29]. This suggests that the Moroccan VOO production needs to be improved in order to provide consumers with higher quality products in favor of their health.

The heat map and the principal component analysis allowed us to classify the provinces studied in terms of VOO chemical composition, representing a valuable method for the characterization of samples of different regions. These preliminary results could be a starting point for a further investigation to be applied to a more consistent number of commercial VOO (and EVOO) samples from *Picholine marocaine.*

## Figures and Tables

**Table 1:** Quantitative values as average (mean ± SD) determination of bioactive molecules in virgin olive oils (VOOs) of *Picholine marocaine* from five provinces studied. Results are expressed as mg.kg^−1^.

Compounds\Region (Provinces)	Fes-Meknes (Taounate)	Drâa-Tafilalet (Errachidia)	Tanger-Tetouan-Al Hoceïma (Chefchaouen)	Fes-Meknes (Taza)	Beni Mellal-Khenifra (Beni Mellal)
α-tocopherol	100.4 ± 0.14	55.8 ± 0.10	55.3 ± 0.27	38.4 ± 0.10	39.9 ± 0.24
α-tocotrienol	<LOQ	<LOQ	2.92 ± 0.07	1.97 ± 0.05	7.60 ± 0.04
β-tocopherol	2.1 ± 0.07	1.9 ± 0.07	2.1 ± 0.06	1.6 ± 0.07	1.7 ± 0.05
γ-tocopherol	5.2 ± 0.06	4.2 ± 0.05	4.5 ± 0.08	4.1 ± 0.10	4.3 ± 0.06
δ-tocopherol	<LOD	<LOD	<LOD	<LOD	<LOD
Tyrosol	<LOD	<LOD	300.6 ± 11.26	<LOD	186.2 ± 2.58
Hydroxytyrosol	6.4 ± 0.54	<LOD	40.0 ± 1.46	2.8 ± 0.15	4.6 ± 0.07
Caffeic acid	<LOQ	<LOD	<LOD	<LOD	3.5 ± 0.06
Ferulic acid	<LOD	4.4 ± 0.43	3.9 ± 0.07	2.0 ± 0.03	0.8 ± 0.04
10-hydroxy-oleuropein aglycone ^a^	25.4 ± 0.09	1.2 ± 0.18	65.7 ± 3.81	0.3 ± 0.00	1.4 ± 0.24
Oleocanthal ^a^	<LOD	<LOD	<LOQ	<LOQ	0.8 ± 0.00
Luteolin	<LOQ	<LOQ	6.4 ± 0.19	7.8 ± 0.72	6.3 ± 0.13
Oleacein	3.1 ± 0.15	0.2 ± 0.02	33.3 ± 1.91	2.3 ± 0.01	3.5 ± 0.17
Apigenin	<LOQ	<LOQ	<LOQ	<LOQ	6.1 ± 0.00
Oleuropein aglycone ^a^	231.3 ± 4.45	1.9 ± 0.00	198.3 ± 13.17	18.6 ± 0.00	20.6 ± 1.24
Luteolin glucoside	2.5 ± 0.03	1.1 ± 0.51	1.5 ± 0.03	3.9 ± 0.22	5.5 ± 1.79
Elenolic acid	31.3 ± 2.00	8.7 ± 0.81	85.6 ± 2.67	13.7 ± 0.26	19.6 ± 1.63
Ligstroside aglycone ^b^	57.3 ± 2.92	0.7 ± 0.04	21.4 ± 0.39	1.2 ± 0.07	3.9 ± 0.19
Acidity%	1.3%	2.0%	2.0%	2.0%	1.1%
∑Vitamin E	107.6 *	61.9	64.8	46.1	53.5
∑Phenolic alcohols	6.4	<LOD	340.6	2.8	190.8
∑Phenolic acids	31.3	13.1	89.5	15.8	24.0
∑Secoiridoids	314.0	3.9	285.4	20.1	26.6
∑Flavonoids	2.5	1.1	7.9	11.7	18.0
∑Phenols	354.2 **	18.1	723.4 **	50.4	259.4 **

Quantitative determination carried out according to the following standard compounds: ^a^ oleuropein; ^b^ verbascoside; * values reported are above 90 mg kg^−1^ complying with Regulation 432/2012 of European Union [27]; ** values reported are above 250 mg kg^−1^ complying with the EU Health Claim [28].

**Table 2:** Average content of fatty acid methyl ester extractions (FAMEs) in VOOs of Picholine marocaine collected from the five provinces studied.

Fatty Acid	Mass Spectral Similarity (%)	Experimental LRI	Tabulated LRI	Taounate (%)	Errachidia (%)	Chefchaouen (%)	Taza (%)	Beni Mellal (%)
Palmitic acid (C16:0)	95	1600	1600	9.99	9.63	8.60	7.66	7.75
Hypogeic acid (C16:1n9)	91	1605	1605	0.31	0.33	0.32	0.32	0.37
Palmitoleic acid (C16:1n7)	96	1615	1616	0.65	0.61	0.47	0.32	0.33
Margaric acid (C17:0)	90	1694	1694	0.03	0.04	0.04	0.04	0.04
Margaleic acid (C17:1n7)	91	1707	1711	0.05	0.05	0.05	0.06	0.06
Stearic acid (C18:0)	95	1802	1801	2.08	2.95	2.42	2.35	2.49
Oleic acid (C18:1n9)	91	1812	1808	75.89	69.79	76.94	79.39	78.15
Cis-vaccenic acid (C18:1n7)	96	1816	1816	2.80	2.33	2.32	2.31	2.19
linoleic acid (C18:2n6)	95	1839	1838	6.71	12.67	7.41	6.25	7.29
α-linolenic acid (C18:3n3)	96	1883	1883	0.83	0.93	0.80	0.67	0.69
Arachidic acid (C20:0)	94	1999	2000	0.27	0.28	0.27	0.26	0.26
Eicosenoic acid (C20:1n9)	91	2014	2015	0.26	0.31	0.28	0.28	0.30
Behenic acid (C22:0)	92	2199	2201	0.07	0.06	0.06	0.07	0.06
Legnoceric acid (C24:0)	90	2400	2400	0.04	0.03	0.03	0.02	0.03

All VOOs complied with Consiglio Oleicolo Internazionale (COI), 2001 [29].
